# Innovative e-Learning Training Modules to Improve Animal Welfare during Transport and Slaughter of Pigs: A Pretest–Posttest Study to Pre-Evaluate the General Didactical Concept

**DOI:** 10.3390/ani13233593

**Published:** 2023-11-21

**Authors:** Rudi Isbrandt, Nina Langkabel, Marcus G. Doherr, Sebastian Haase, Diana Meemken

**Affiliations:** 1 Working Group Meat Hygiene, Institute of Food Safety and Food Hygiene, School of Veterinary Medicine, Freie Universität Berlin, Königsweg 67, 14163 Berlin, Germany; 2 Institute for Veterinary Epidemiology and Biostatistics, School of Veterinary Medicine, Freie Universität Berlin, Königsweg 67, 14163 Berlin, Germany; 3 School Pedagogy and School Improvement Research, Department of Education and Psychology, Freie Universität Berlin, Habelschwerdter Allee 45, 14195 Berlin, Germany

**Keywords:** education, online training, slaughterhouse, abattoir, pig, animal well-being, didactic, knowledge test

## Abstract

**Simple Summary:**

Legal regulations at European and national level create the basis for the protection of animals. A certificate of competence is obligatory when animals intended for human consumption are handled or slaughtered. Within the project “eSchulTS2”, e-learning training courses for employees of transport companies and abattoirs were developed to the improve animal welfare of pigs during transport and slaughter. This study was to assess the impact on the respective knowledge of employee groups with different educational level and background at one pig abattoir in Germany and to pre-evaluate the innovative and low-barrier didactical concept of the e-learning training courses. By using questions of knowledge, it was shown that more questions of knowledge were answered correctly after conducting the courses. Together with interactions shown by further statistical methods, this pre-evaluation showed that with the underlying didactical concept, an increase in knowledge was achieved.

**Abstract:**

In addition to the information on the possession of a certificate of competence, there are no concrete obligations for repetitive training for personnel handling live animals at transport and slaughter. Deficiencies in the animal-welfare-friendly handling of pigs are known. The developed pilot modules “Handling of pigs” and “Electrical stunning” were tested in a pretest–posttest study in German and Romanian using questions of knowledge before and after the implementation of the modules. In this study, 45 and 46 datasets of participants could be analyzed. The mean percentages of correctly answered questions in the posttest increased by 5.6% in the module “Handling of pigs” and by 10.6% in the module “Electrical stunning”. A significant interaction was found for the language match and trend categories in the module “Handling of pigs”. No Romanian native speaker had a positive trend in this module. For both modules separately, participant education level significantly interacted with the language match and the presence or absence of a certificate of competence. Comparing the percentages of the correct given answers, significant interactions in the subgroups were more common in the module “Electrical stunning”. One question in “Electrical stunning” was correctly answered significantly more often in the posttest. Because of the positive mean trends of knowledge within this pre-evaluation, we assume the didactical concept was suitable for our target groups. Holders of a certificate of competence also gave more correct answers in the post-test. This underlines the importance of repetitive training. Differences in the trends of knowledge gain seem to be topic and experience related.

## 1. Introduction

### 1.1. Animal Welfare during Transport and Slaughter

Welfare of livestock intended for slaughter is in the interest of consumers, who are increasingly concerned about animal welfare in intensified animal husbandry systems [1]. Despite the increasing numbers of animals that abattoir personnel have to care for, positive human–animal contact is important, and therefore, knowledge of how to correctly handle animals is required [2]. The social and political importance of animal welfare is reflected in European and German legislation dealing with the transport and slaughter of animals intended for human consumption. The German Animal Welfare Act explicitly states that no one may inflict pain, suffering, or harm on an animal without reasonable cause [3]. The necessity for authorization of transporters and the establishment of a certificate of competence for personnel transporting animals is defined in Regulation (EC) No 1/2005 on the protection of animals during transport and related operations [4]. Further topics and more specific information, such as recognition and withdrawal of the certificate of competence, are addressed in the German national law on the transportation of animals [5]. The certificate of competence, which personnel working at the abattoir with live animals or slaughtering them also have to hold, and the conditions under which it is issued are addressed in Regulation (EC) No 1099/2009 on the protection of animals at the time of killing [6]. The German national law on the protection of animals during slaughter specifies the competences needed, the content of teaching units, and the conduct of examinations to receive this certificate. A certificate of competence is valid for an unlimited period, but can be withdrawn if the requirements laid down in Regulation (EC) No 1099/2009 are violated several times [7]. The need for repeated training regarding animal welfare at the day of slaughter is not further addressed in European or German national law. However, in some German districts, repetitive training courses on animal welfare are implemented [8]. The German official Working Group for Consumer Protection recommends that training is regularly conducted in order to sustain the level of knowledge of employees working with animals [9]. The responsibility to conduct training repeatedly can lie with the animal welfare officers of larger abattoirs, since they have to guarantee that all employees know and understand the standard operating procedures [10].

### 1.2. Preliminary Work and Approach to Identify Suitable Training Content

The project “Development of target group-specific e-learning modules to improve animal welfare during transport and slaughter of cattle and pigs” (Acronym: eSchulTS2) was mainly implemented by members of the Freie Universität Berlin, School of Veterinary Medicine: Institute of Food Safety and Food Hygiene, Working Group Meat Hygiene, Institute of Animal Welfare, Animal Behavior and Laboratory Animal Science and Institute for Veterinary Epidemiology and Biostatistics. The core team was supported by the Department of Education and Psychology, Further Education and Educational Management of Freie Universität Berlin as well as by an industry partner.

The bases for preparing the training courses for pigs were a questionnaire on the status quo of animal welfare training at German and Austrian abattoirs [11] and the results of a systematic literature review to find out the “Impact of procedures and human–animal interactions during transport and slaughter of pigs” [12]. After conducting a Delphi-type expert elicitation in which experts rated the potential impacts, the topic areas for the final e-learning training modules were set [13]. Initially, the two training modules, “Handling of pigs” and “Electrical stunning”, were designed to assess the didactical and technical concept of the e-learning modules with the potential to refine and finalize the concept. At the end of the project, a total of seven e-learning training modules in the following courses will be provided online: Handling of pigs; Fitness for transport; Stunning procedure (including Electrical-stunning; Stunning with carbon dioxide; Check of stunning effectiveness; Post-stun with captive bolt); and Bleeding. All e-learning modules will be available in multiple languages and will be free of charge for all interested users.

### 1.3. Didactical Concept for the e-Learning Training Courses

**Figure 1:**
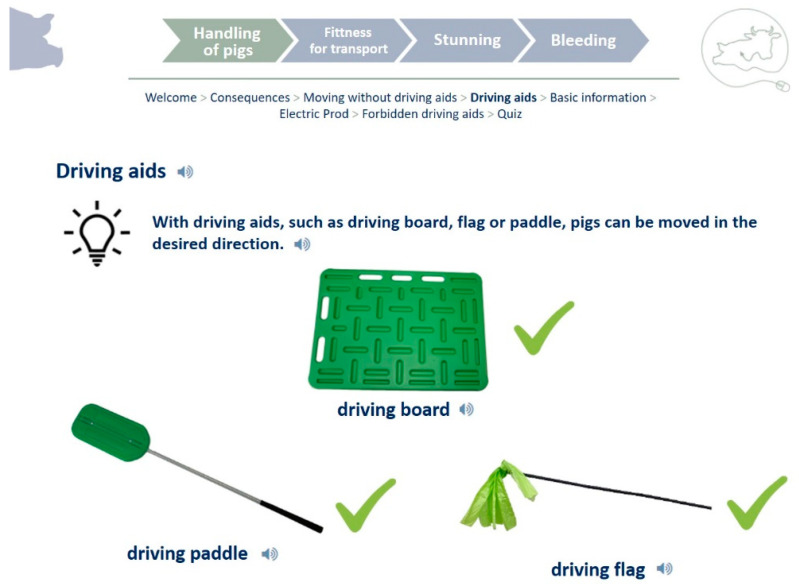
Screenshot from website “tet.folio”, module “Handling of pigs”, topic “Driving aids”; translated for publication.

In the final e-learning courses, each participant can choose between the languages German, Romanian, Polish, Hungarian, Bulgarian, and English. The didactical concept is based on clear structures and a specific color scheme for each course, which ensures easy orientation. The minimum use of text components and use of simple language helps the participants to focus on the content of the modules. These techniques are how content and visual overload, especially for low-qualified participants, is avoided. A table of contents is always visible and also has the function of a progress bar (Figure 1).

The basis for communication of information is combinations of pictures and symbols. Videos with spoken text (voiceover) serve as the central knowledge transfer instrument in order to also include illiterates. The voiceover is available in the different selectable languages, which, apart from English, have been proven to be the most common mother languages of employees in abattoirs in German-speaking countries [11]. Selectable information boxes provide knowledge beyond the basic training content that is especially relevant for animal welfare officers, official veterinarians, or other interested participants. It is possible to repeat individual parts of the modules and videos or the entire modules at any time. An estimated working time of not more than 15 min for each module is intended to maintain concentration and to prevent the fatigue of participants. At the end of each module, participants can assess their own learning success by completing a quiz. A certificate of participation can be printed on request. Our aim was to pre-evaluate the underlying didactical concept of two e-learning modules with a pretest–posttest study design before elaborating on the other modules. This pre-evaluation was carried out by identifying short-term changes in knowledge at the level of individual participants as well as within groups of participants with similar demographic characteristics. In addition, we wanted to identify potential influences on different knowledge trends among participant groups, at the single-question level and between the two modules. The experience gained during this pre-evaluation process can also be incorporated into the intended final evaluation of all modules. The evaluation will follow the same approach and will be complemented by an additional posttest after a longer period of time to analyze the long-term gain of knowledge. This gives the opportunity to eliminate weaknesses and to adopt strengths of the methodology in the future.

To the best of the authors’ knowledge, there are no comparable training materials available in Europe to improve animal welfare that were specifically developed for employees of pig transport and abattoir companies that have a limited education background and that lack (German) language proficiency.

## 2. Materials and Methods

### 2.1. Method Used and Participants Included

We used the method of pretest–posttest design [14] to evaluate the two e-learning training modules “Handling of pigs” and “Electrical stunning”. In this design, questions to test knowledge were asked at two timepoints, directly before (pretest) and directly after (posttest) completion of the respective modules, to measure the short-term increase of knowledge.

The questions were designed especially for knowledge testing within the frame of developing and evaluating the concept of our e-learning training modules; as such, they will not be included in the final e-learning platform for all participants. At the time of this evaluation, the training modules were available only in German and Romanian. Participants with other mother tongues carried out the training in German. It was previously established by the animal welfare officer that they speak and understand German sufficiently well to very well. Six easy understandable questions were asked for the module “Handling of pigs” and five questions were asked for the module “Electrical stunning” (Supplementary Material File S1). For each question, three answer options (the correct answer and two distractors) were provided. There was the possibility to select between none, one, two, and all answers, but the given answers were rated as correct only when the correct answer was chosen. Participants were not told that only one answer option was correct. The same questions were asked both directly before conducting and directly after completing the module. The order of questions and the respective answer options were randomized for every participant and timepoint. After answering, the participants were not told if they had chosen the correct answer, in order not to influence the posttest.

The e-learning training courses were tested and pre-evaluated in one of the industry partner’s pig abattoirs, where a computer pool room was available. Therefore, all participants were employed by the industry partner. Stunning was performed with carbon dioxide. Necessary emergency slaughters were performed after manual electrical stunning, whereas re-stunning was also performed with the captive bolt. The e-learning training courses and tests were conducted in September and October of 2022. It was not necessary for the participants to have specific prior knowledge or a certificate of competence, which also means that employees not handling live animals were allowed to take part. The group of employees without a certificate of competence should represent newcomers in this field currently undergoing training to receive a certificate of competence in the near future. The pretests and posttests were implemented in the e-learning platform “tet.folio” of Freie Universität Berlin, and made available online. Individual login details were generated that allowed us to link the responses from the pretest and posttest assessments for each person. The on-site implementation of the tests and supervision was within the responsibility of the local animal welfare officer of the abattoir. Participants, either in groups of up to ten persons in a computer pool room or individually in an office room, were given a standardized introduction by the animal welfare officer. The animal welfare officer was in the room the entire time and made sure that the participants did not communicate with each other. Furthermore, the officer was not allowed to help the participants if they had technical questions or if they had questions about the module content. This should simulate the aimed final e-learning situation in order to show whether the platform is intuitive to use. However, questions and issues were noted and shared by the animal welfare officer with the project team for further improvements of the modules and platform settings. The participants’ demographic data (country of origin, education, working position at the abattoir, and information regarding certificate of competence) were linked pseudonymously to the login details by the animal welfare officer and made available only to the project team for the purpose of analysis. The identity of the participants was not disclosed to the project team, and the individual participant’s performance was not shared with the animal welfare officer or the employer.

### 2.2. Statistical Analysis

Responses were collected in “tet.folio” and downloaded as an MS Excel file (MS Office LTSC Professional Plus 2021, MS Excel Version 2108). The linked demographic data were received in a separate Excel file and merged with the response information through the internal identifier (login-ID). For the various subgroup analyses, participants were categorized regarding possession of a certificate of competence for transporting/handling/slaughtering animals (present or absent), and the training effect was compared between these two groups. In addition, the association between language match (module in mother tongue available or not; estimated on the basis of the records of the animal welfare officer regarding the participants’ countries of origin) and training success was analyzed. Educational background was categorized based on the personal educational level (education level): (A) secondary school education, (B) secondary school education and professional training, (C) tertiary education. In another dimension, categories were defined by participants with specialization (education specialization): (A) secondary school education, (B) secondary school education and professional training in the field or tertiary degree in the field (of slaughter, animal husbandry, veterinary medicine, etc.), and (C) secondary school education and professional training in another field or tertiary degree in another field of competence. Another group of analyses was used to assess the change in averages of the individual participant’s proportions of correct answers (given in percent) between the two time points. Here, only those questions were included for which an answer was actually provided.

For the last set of analyses, each participant’s trend of changing knowledge (answer status correct or not before and after training) was defined and, therefore, we determined whether each participant (+) improved, (=) remained the same or (−) worsened. We defined this clustering of trends into three types, as trend-category-3. However, because of the small number of participants and to ensure better statistical results, we reduced this to a trend-category-2. Hence, we also used two categories “same or worse” (combination of the categories “=” and “−“ from variable trend-category-3) and “better” (+) in our analysis.

Descriptive statistics were generated in MS Excel. The average percentages of correct answers were calculated overall and for participants in defined subgroups. Further analyses were carried out with IBM SPSS Statistics version 28 for windows. In addition to the description of frequencies (for categorical data), we tested the independent variables with the Fisher–Freeman–Halton exact test via cross tables to identify possible influences of the demographical subgroups (regarding certificate of competence, education, and language match) on the trend categories. In addition, we crossed the subgroups among themselves, also with the Fisher–Freeman–Halton exact test.

By using the Wilcoxon signed rank test for dependent repeated samples, the values (over all participants) of the average percentages of correct answers given at the two timepoints (before and after training) were analyzed overall and within each of the subgroups (certificate of competence, mother tongue, education level, and education specialization).

Cross-tables and the McNemar test statistic were used to assess the influence of training results at the single-question level (right or wrong answer given) in the pretest and posttest.

The knowledge testing was approved by the ethics committee of Freie Universität Berlin under ZEA Nr. 2022-016.

## 3. Results

### 3.1. Participants

In total, datasets of 45 participants in the module “Handling of pigs” were included for statistical analysis. The module “Electrical stunning” was performed by 46 participants. Demographical data regarding education or educational level were missing for two participants in the module “Handling of pigs” and for an additional participant in the module “Electrical stunning” (n = 3). As the questions were not compulsory, not all participants selected an answer to every question.

### 3.2. Overall Knowledge Gain

**Figure 2:**
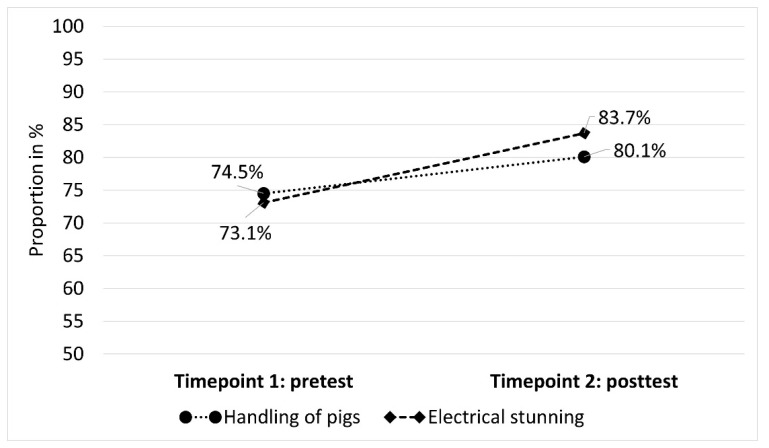
Average percentage of correct answers given in the pretest and posttest.

After calculating the average percentage of correct answers for the pretest and posttest assessment over all participants, it turned out that the percentage of correctly answered questions was higher in the posttest phase in both modules. The percentage of correct answers increased from pretest to posttest by 5.6% in the module “Handling of pigs” and by 10.6% in the module “Electrical stunning” (Figure 2).

In the module “Handling of pigs”, 7 out of 45 participants (15.6%) worsen, 26 (57.8%) had the same, and 12 (26.7%) had better results in the posttest compared to the pretest (Table 1). In the module “Electrical stunning”, 4 out of 46 participants (8.7%) achieved worse results, 19 (41.3%) the same, and 23 (50.0%) better results in the posttest than in the pretest (Table 2).

### 3.3. Subgroup Analysis: Certificate of Competence, Language Match, Education

In the following, only some general results from the subgroup analysis are presented. The overview is shown in Table 1, Table 2 and Table 3.

About three quarters of the participants currently hold a certificate of competence. Around half of the participants could choose their mother tongue German in the modules, while the choice of Romanian as mother tongue was possible for about 30% of the participants (Table 1 and Table 2). For both modules separately, the group of participants for whom the level of education was known was the same.

When examining the presence or absence of a certificate of competence in combination with the knowledge trends (trend-category-3 and trend-category-2), no statistically significant associations were detected for each of the modules. In the module “Handling of pigs”, 29.4% of participants with a current certificate of competence had better results in the posttest, i.e., they answered more questions correctly) than in the pretest. Within the module “Electrical stunning”, it was noticeable that no participant without a certificate of competence obtained worse results in the posttest compared to the pretest, and the majority obtained better results (63.6%).

In the e-learning module “Handling of pigs”, the possibility to choose one’s own mother tongue significantly interacted with both trend-category-3 (*p* = 0.031, Fisher–Freeman–Halton exact test) and trend-category-2 (*p* = 0.016; Fisher–Freeman–Halton exact test). No significant interaction was found in the module “Electrical stunning”. Concerning the selection of the mother tongue in the “Handling of pigs” module, no participant who could chose Romanian as the mother tongue had a positive knowledge trend (Table 1 and Table 2). In the “Electrical stunning” module, 28.6% of the participants who chose Romanian as their mother tongue achieved a positive knowledge trend. This percentage was around half of the percentage of those who chose German as their mother tongue (61.9%) and those who were not able to choose their mother tongue (54.5%).

Regarding education, the mean trends were similar within the groups “education-level” and “education-specialization” for the modules “Handling of pigs” and “Electrical stunning”. In both modules, the choice of their own mother tongue as the module language significantly interacted with the category education level (*p* = 0.042; Fisher–Freeman–Halton exact test). Participants who chose Romanian as their mother tongue had nearly all secondary school education and completed professional training (90.9%; 10/11); the other participant had secondary school education only (9.1%; 1/11). None of the Romanian speakers had a tertiary degree. When choosing German as mother tongue was possible, one participant had secondary school education only (4.8%; 1/21), whereas twelve participants had secondary school education and completed professional training (57.1%; 12/21) and eight had a tertiary degree (38.1%; 8/21). A slightly different distribution of the educational levels was seen for participants who could not choose their mother tongue (graduation only: 27.3% (3/11); graduation and professional training: 54.5% (6/11); graduation and studies: 18.2% (2/11)). The three participants with only secondary school graduation were from Russia (n = 1) and Turkey (n = 2). Participants with additional professional training came from Greece (n = 3), Poland (n = 2), and Bulgaria (n = 1) and previously worked as carpenters, tilers, electricians, locksmiths, or butchers. Participants with a tertiary degree came from Greece (n = 1) and Poland (n = 1) with former jobs as teachers.

For both modules, the presence or absence of a certificate of competence significantly interacted with the category education level (*p* = 0.015; Fisher–Freeman–Halton exact test). All participants with only secondary school graduation had a certificate of competence (100.0%; 5/5). From the 28 participants with secondary school education and professional training, the majority held a certificate of competence (82.1%; 23/28) and five did not (17.9%). The distribution was different among participants with a tertiary education, where the certificate of competence was held by four participants (40.0%; 4/10), but was not held by six (60.0%; 6/10), respectively. A significant interaction was also found for the presence or absence of the certificate of competence and the language match in both modules separately (“Handling of pigs” *p* = 0.02; Fisher–Freeman–Halton exact test/“Electrical stunning” *p* = 0.001; Fisher–Freeman–Halton exact test). Of the participants without a certificate of competence, ten were German and one originated from Poland. Participants with a certificate were equally distributed. Eleven of these participants were German, thirteen and fourteen of these participants in the modules “Handling of pigs” and “Electrical stunning”, respectively, were Romanian, whereas ten participants without certificates originated from other countries.

Crossing education subgroups and trend categories from the two modules, a significant interaction was found only in the “Electrical stunning” module for education specialization and trend-category-3 (*p* = 0.05; Fisher–Freeman–Halton exact test). Only participants with a secondary school education and professional training or tertiary degree from another field showed worse results in the posttest (12.0%), although more in this group had the same (28.0%) or better results in the posttest (60.0%). Participants with a secondary school education and professional training or tertiary degree in the field had the same results (76,9%) or obtained better results (23.1%). Participants with a secondary school education only had the same results (40.0%) or a positive trend (60.0%) in the posttest.

When comparing the percentages of correct given answers in the pretest and posttest, significant associations were found for both modules (Table 3).

### 3.4. Analysis on Single-Question Level

**Figure 3:**
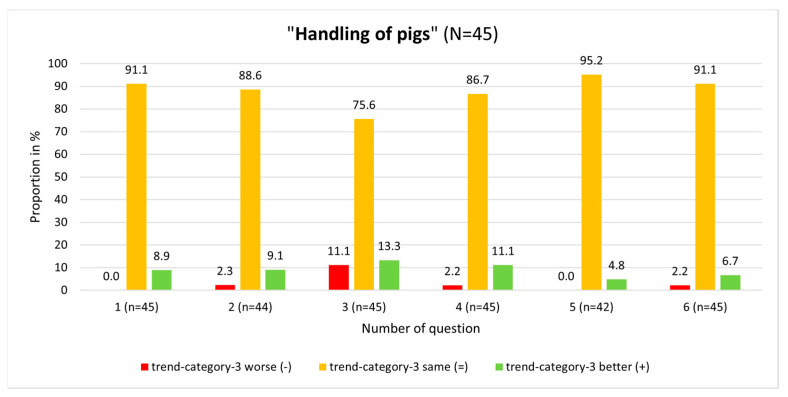
Results on single-question level in relation to trend-category-3 in the module “Handling of pigs”.

At the single-question level in the “Handling of pigs” module, a great variety regarding trend-category-3 was seen for question No. 3 (Figure 3). In this module, question No. 3 produced the greatest positive and negative trends. Also, at the single-question level, participants produced better results (i.e., have given more correct answers) in the “Electrical stunning” module than in the other module.

**Figure 4:**
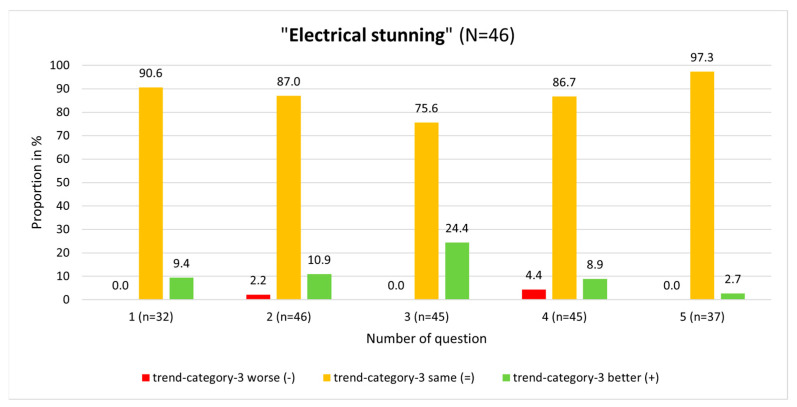
Results on single-question level in relation to trend-category-3 in the module “Electrical stunning”.

In the “Electrical stunning” module, participants’ knowledge was worse in the posttest compared to the pretest in only two questions. The participants achieved worse results in four questions in the “Handling of pigs” module (Figure 3 and Figure 4). Comparing the pretest and posttest results for question No. 3 (“How many seconds must the minimum current be held so that safe stunning is achieved in fattening pigs?”) in the “Electrical stunning” module, a strong significant interaction was found (*p* < 0.001; McNemar test). On answering this question, 24.4% of the participants produced a positive knowledge trend (so gave wrong answers in the pretest, but correctly answered in the posttest), which was the highest among all questions (Figure 4).

## 4. Discussion

### 4.1. Study Design and General Limitations

In pretest–posttest studies, a group of participants perform the same test before and after an educational intervention [14]. This study type is often used in the evaluation of medical and management training [15,16]. A general criticism of one-group pretest–posttest designs is that this study type does not have a comparative group [17]. In order to include the highest possible total number of participants for our study, we used the single-group pretest–posttest design. In addition, it was difficult to reach a larger number of participants for this pre-evaluation due to the specific study topic and the generally difficult access to abattoir employees. However, the division into subgroups, each with a resultant relatively small participant number, hampered our statistical analysis and descriptive statistics, because spikes could result from each subgroup’s small number of participants. Afterwards, the subgroups were compared with each other. Further interpretations have been refrained from in order to avoid overinterpretation of the underlying data. In hindsight, we should have set all questions as obligatory, thereby making participants answer all of them. This could have shown more deficits in knowledge and, therefore, could have impacted the results. The small number of knowledge questions in the pre- and posttests reflected only a part of the information imparted in the modules. In order to minimize the dropout rate and increase motivation, we decided that the module tests would both contain five to six questions. We consider this number of questions sufficient for a general statement on the didactical concept used in this pre-evaluation study.

### 4.2. Overall Knowledge Gain

The increased mean percentages of correct answers at the posttest showed that the participants gained knowledge during the e-learning lectures in both modules, at least in the short term. Therefore, we suppose that the didactical concept used was suitable for our target groups. As shown by the frequencies of participants worsening or improving in the posttest compared to the pretest, there were no differences between the modules. The module “Handling of pigs” reflects people’s day-to-day work with pigs and does not have as many questionable facts as the “Electrical stunning” module. In our opinion, however, the questions in the modules were about equally difficult. Although there were some more complex questions on the use of the electrical prod in the module “Handling of pigs”, the correct answers in general were communicated well in the module, in written words, within pictures and in the videos with voiceovers. This could be due to the fact that employees were already sensitized to the use of this driving aid after gaining a certificate of competence or taking part in other trainings. A reason for the higher positive learning trend in the module “Electrical stunning” may be the fact that in the abattoir where the study took place, stunning with carbon dioxide is used as the standard method. That means that electrical stunning is not part of the day-to-day work and is only used for emergency stunning or killing. Nevertheless, facts about this stunning device should be known by the employees who perform stunning. Reasons for the higher positive knowledge trend resulting from the “Electrical stunning” module could be that in general, more knowledge was imparted, and more animal welfare-related facts are necessary when performing electrical stunning. 

It is known from other studies that most facts are forgotten in the first hours after memorization [18] and also, that repetition strengthens the retrieval of information [19]. Moreover, practice and repetition promote storage in the long-term memory [20]. These facts can be reasons for the positive learning trend found after knowledge testing directly after conducting the modules. Another possibility to be mentioned is that the increase in correct answered questions could be by chance. For the final evaluation of the modules with regard to long-term memorization, it will be interesting to ask the same questions again in a follow-up test after a few days or weeks. For our aim of a pre-evaluation of the general didactical concept, though, the positive trend and overall failures were more of interest and the improvements will be implemented in the pre-final versions of the modules. Furthermore, analyses of the data will focus on participants with the same learning trend. Care must be taken when interpreting the data as they are paired data. The influence of the individual question level is not considered further. The authors wanted to give a general overview.

### 4.3. Subgroup Analysis: Certificate of Competence, Language, Education

Since it was not our aim to test the e-learning training modules only with persons who held a certificate of competence, we also enrolled participants without one. Because of legal requirements, all employees that have contact with living animals at the abattoir must hold the certificate before they are allowed to transport, move, stun, or kill animals, or check the stunning effectiveness [4,6]. This is reflected by the high number of participants with a certificate of competence. This fact may be the reason for a relatively high knowledge level in the pretest and resulting knowledge trends with a consequently flatter increase in the “Handling of pigs” module. For subsequent evaluations, participants with a certificate of competence should be asked whether they are currently working with live animals or not. This could provide new findings with regard to the subgroups and the interpretation of the didactical concept. 

In this study, the countries of origin of the participants who were not from Germany or Romania were in line with the results of our former study in which we asked for the countries of origin of the employees of 29 slaughter companies in Germany and Austria [11]. Surprisingly, participants who could choose Romanian as their mother tongue had worse results than the other language subgroups. The reason for this effect remains unclear. The existing career changing in migration workers [21] might be one speculative reason for the worse results we detected in Romanian-speaking participants, as semi-trained and untrained employees have fewer basic skills [22]. It could be that the Romanian employees in our study represent more a group of career changers, and the results are not related to the country of origin per se. A comparison with another group of career changers to date has not been possible, because the modules have only been available in German and Romanian. In addition, a high impact of individual results on the group result for this language group could have occurred because of the relatively small number of Romanian native speakers who participated. 

All participants had at least completed a secondary school education. This is in accordance with the results of our previous survey, in which over 30% of the participants estimated that 80–100% of the employees have a secondary school education [11]. We suppose that participants with a secondary school education more likely work in animal welfare sensitive areas, because all of them had a certificate of competence. In contrast, fewer participants with a tertiary education had a certificate of competence and could work in other areas within the abattoir. A reason for this could be that the slaughter industry has a lack of skilled workers [21], and in Germany, many people working in that industry are career changers originating from other European countries [23]. This is supported by the fact that teachers from other countries were also employed in the animal welfare sensitive areas of the abattoir where the study took place.

By comparing the percentages of the correct given answers in the pretest and posttest, a greater number of significant interactions were found in the module “Electrical stunning”. This finding supports the fact that the trends in knowledge change were influenced by and related to the individual module topic.

### 4.4. Analysis on Single-Question Level

Based on the result for question No. 3 “Which statement about moving pigs is correct?“ in the module “Handling of pigs” (Supplementary Material File S1), it can be seen that the presentation of information in the module videos and the question in the knowledge test can influence the result. To correctly answer this question, the participants had to mentally transform a negative statement from a video into a positive answer from the question. In addition, the relatively long answers, which are generally not recommended [24], could have had an impact, too. The wrong answer, “In a calm environment, pigs run slower in the desired direction because they explore everything curiously,” was given often. For us, this answer was not surprising as we have observed that people who move pigs often interact a lot with them. In our experience, employees often think that pigs have to be stressed that they start to move or move in the desired direction. As a consequence, this fact should be addressed more during hands-on training in the future so that employees understand a calm environment as positive for the animals and the workflow. The question, “Which statement about moving pigs is correct?”, could be judged as a “best answer” question and not a clear “right or wrong” question [24], which could have made the question more difficult. The way the questions were asked and answers were provided in relation to what was taught was shown to have an influence. This will be changed accordingly in the updated version of the modules in the questions for the final evaluation. The high percentage of participants improving in question No. 3 at the posttest also affected the mean trend in the module “Electrical stunning” because of the few questions asked.

## 5. Conclusions

In both e-learning training modules, “Handling of pigs” and “Electrical stunning”, a knowledge gain was shown. The mean knowledge trend differed between the modules. The knowledge trend seems to be topical and educational and therefore experience related. No clear associations were identified between knowledge trends and whether or not participants held a certificate of competence for animal welfare sensitive areas. The influence of the possibility to choose training in one’s own mother tongue is not clear. We speculate that the factor of career changing by Romanian speaking participants might be a reason for their worse results compared to the other participants. The fact that an increase in knowledge was also seen among participants with a certificate of competence underlines the importance of repetitive training courses in animal welfare topics. The design of the questions in relation to the knowledge taught in the modules can have an influence on the given answers. Because of the positive overall knowledge trend, we interpret the general didactical concept with intuitive color schemes, simple language, little text, voiceover videos, and easily understandable animations as suitable for the included target groups.

## Figures and Tables

**Table 1:** Results of demographical data, trend-category-3, and means of correct answers in the module “Handling of pigs”.

	n/N %	Trend-Category-3: Individual Knowledge Trends	Mean of Correct Answers in Pretest in %	Mean of Correct Answers in Posttest in %	Mean of Trend (Change from Before to After) in %
all participants	45/45 100.0%	− 15.6% (7/45) = 57.8% (26/45) + 26.7% (12/45)	74.5	80.1	+5.6
**Certificate of competence**					
yes, hold a certificate of competence	34/45 75.6%	− 17.6% (6/34) = 52.9% (18/34) + 29.4% (10/34)	73.9	79.7	+5.8
no certificate of competence	11/45 24.4%	− 9.1% (1/11) = 72.7% (8/11) + 18.2% (2/11)	79.5	84.6	+5.1
**Selection of mother-tongue**					
German possible	21/45 46.7%	− 14.3% (3/21) = 52.4% (11/21) + 33.3% (7/21)	74.5	80.1	+5.6
Romanian possible	13/45 28.9%	− 30.8% (4/13) = 69.2% (9/13) + 0.0% (0/13)	74.1	79.0	+5.0
not possible	11/45 24.4%	− 0.0% (0/11) = 54.5% (6/11) + 45.5% (5/11)	71.0	80.6	+9.6
**Education-level**					
secondary school education	5/43 11.6%	− 0.0% (0/5) = 40.0% (2/5) + 60.0% (3/5)	73.8	79.6	+5.8
secondary school education and professional training	28/43 65.1%	− 21.4% (6/28) = 57.1% (16/28) + 21.4% (6/28)	74.5	80.1	+5.6
tertiary education	10/43 23.3%	− 0.0% (0/10) = 70.0% (7/10) + 30.0% (3/10)	75.6	82.8	+7.2
**Education-specialization**					
secondary school education	5/43 11.6%	− 0.0% (0/5) = 40.0% (2/5) + 60.0% (3/5)	73.8	79.6	+5.8
secondary school education and professional training in the field or tertiary degree in the field	13/43 30.2%	− 23.1% (3/13) = 61.5% (8/13) + 15.4% (2/13)	73.9	79.7	+5.8
secondary school education and professional training in another field or tertiary degree in another field	25/43 58.1%	− 12.0% (3/25) = 60.0% (15/25) + 28.0% (7/25)	74.3	79.7	+5.4

“−“: worse/negative trend; ”=”: same/no trend; “+”: better/positive trend.

**Table 2:** Results of demographical data, trend-category-3, and means of correct answers in the module “Electrical stunning”.

	n/N %	Trend-Category-3: Individual Knowledge Trends	Mean of Correct Answers in Pretest in %	Mean of Correct Answers in Posttest in %	Mean of Trend (Change from Before to After) in %
all participants	46/46 100.0%	− 8.7% (4/46) = 41.3% (19/46) + 50.0% (23/46)	73.1	83.7	+10.6
**Certificate of competence**					
yes, hold a certificate of competence	35/46 76.1%	− 11.4% (4/35) = 42.9% (15/35) + 45.7% (16/35)	74.0	83.3	+9.3
no certificate of competence	11/46 23.9%	− 0.0% (0/11) = 36.4% (4/11) + 63.6% (7/11)	76.4	88.5	+12.1
**Selection of mother-tongue**					
German possible	21/46 45.7%	− 4.8% (1/21) = 33.3% (7/21) + 61.9% (13/21)	73.1	83.7	+10.6
Romanian possible	14/46 30.4%	− 14.3% (2/14) = 57.1% (8/14) + 28.6% (4/14)	73.9	83.4	+9.5
not possible	11/46 23.9%	− 9.1% (1/11) = 36.4% (4/11) + 54.5% (6/11)	78.8	86.7	+7.9
**Education-level**					
secondary school education	5/43 11.6%	− 0.0% (0/5) = 40.0% (2/5) + 60.0% (3/5)	76.6	85.8	+9.2
secondary school education and professional training	28/43 65.1%	− 10.7% (3/28) = 50.0% (14/28) + 39.3% (11/28)	73.1	83.7	+10.6
tertiary education	10/43 23.3%	− 0.0% (0/10) = 30.0% (3/10) + 70.0% (7/10)	81.2	90.0	+8.8
**Education-specialization**					
secondary school education	5/43 11.6%	− 0.0% (0/5) = 40.0% (2/5) + 60.0% (3/5)	76.6	85.8	+9.2
secondary school education and professional training in the field or tertiary degree in the field	13/43 30.2%	− 0.0% (0/13) = 76.9% (10/13) + 23.1% (3/13)	74.0	83.3	+9.3
secondary school education and professional training in another field or tertiary degree in another field	25/43 58.1%	− 12.0% (3/25) = 28.0% (7/25) + 60.0% (15/25)	74.6	84.8	+10.2

“−“: worse/negative trend; ”=”: same/no trend; “+”: better/positive trend.

**Table 3:** Comparing the means of correct answers given in pretest and posttest with the Wilcoxon signed rank test.

	Module “Handling of Pigs”			Module “Electrical Stunning”		
	n/N %	Test Statistic Value	*p*-Value	n/N %	Test Statistic Value	*p*-Value
all participants	45/45 100.0%	372.0	0.533	46/46 100.0%	322.0	0.001 *
**Certificate of competence**						
yes, hold a certificate of competence	34/45 75.6%	252.0	0.683	35/46 76.1%	165.0	0.024 *
no certificate of competence	11/45 24.4%	13.0	0.596	11/46 23.9%	28.0	0.018 *
**Selection of mother-tongue**						
German possible	21/45 46.7%	81.0	0.226	21/46 45.7%	104.0	0.001 *
Romanian possible	13/45 28.9%	0.0	0.002 *	14/46 30.4%	12.0	0.750
not possible	11/45 24.4%	40.0	0.036 *	11/46 23.9%	23.5	0.105
**Education-level**						
secondary school education	5/43 11.6%	9.0	0.144	5/43 11.6%	6.0	0.102
secondary school education and professional training	28/43 65.1%	148.5	0.483	28/43 65.1%	82.0	0.063
tertiary education	10/43 23.3%	13.5	0.102	10/43 23.3%	28.0	0.016 *
**Education-specialization**						
secondary school education	5/43 11.6%	9.0	0.144	5/43 11.6%	6.0	0.102
secondary school education and professional training in the field or tertiary degree in the field	13/43 30.2%	26.5	0.554	13/43 30.2%	6.0	0.102
secondary school education and professional training in another field or tertiary degree in another field	25/43 58.1%	120.5	0.556	25/43 58.1%	148.0	0.006 *

* Significant interaction found by comparing the percentage of correct given answers in the pretest to the percentage of correct given answers in the posttest; alpha level of significance = 0.05.

## Data Availability

The datasets that support the findings of this study, with the exception of the published Figures, Tables, and Supplementary Material, are not openly available due to reasons of sensitivity, but are available from the corresponding author upon reasonable request.

## Supplementary Materials

The following supporting information can be downloaded at: https://www.mdpi.com/article/10.3390/ani13233593/s1, File S1: Questions of knowledge for pre- and posttest in the modules “Handling of pigs” and “Electrical stunning”.
