# Supplementary material for: Innovative e-Learning Training Modules to Improve Animal Welfare during Transport and Slaughter of Pigs: A Pretest–Posttest Study to Pre-Evaluate the General Didactical Concept
Source: Animals (Basel). 2023 Nov 21;13(23):3593. doi: 10.3390/ani13233593 (PMC10705144; doi:10.3390/ani13233593)
Supplement: Supplementary file 1 [file animals-13-03593-s001.zip › animals-2702885-supplementary.pdf]

Supplementary Material S1: Questions of knowledge for pre- and posttest in the modules "Handling of pigs" and "Electrical stunning".

Languages during the testing period: German and Romanian, translated into English for publication. Correct answer highlighted in bold.

a) **The following are the questions of knowledge for the modules "Handling of pigs" and "Electrical stunning" in German.**

<u>**Umgang mit Schweinen**</u>

1) Das Treiben von Schweinen kann auch ohne Treibhilfen stattfinden. Hierzu eignen sich
   - **das Ansprechen der Schweine oder ein Klatschen in die Hände**
   - ein leichtes Schlagen mit der Hand in den Rückenbereich der Schweine
   - lautes Schreien und starke Hand- und Beinbewegungen durch die treibende Person

2) Welche Aussage zur elektrischen Treibhilfe ist richtig?
   - **Die elektrische Treibhilfe darf nur 1x angewendet werden, wenn Schweine vor der Vereinzelung zur Betäubung nicht weiterlaufen**
   - Die elektrische Treibhilfe darf in allen Bereichen des Betriebes angewendet werden (Transporter, Wartestall, Zutrieb zur CO2 und Elektrobetäubung), jedoch nur 1x pro Schwein
   - Die elektrische Treibhilfe darf in Ausnahmefällen mehrmals (bis zu 3x) am Schwein angewendet werden, jedoch nur kurz vor der Betäubung

3) Welche Aussage zum Treiben von Schweinen ist richtig?
   - **Das Treiben von Schweinen funktioniert am besten in einer ruhigen und stressfreien Umgebung**
   - Gestresste Schweine laufen besser in die gewünschte Richtung
   - In ruhiger Umgebung laufen die Schweine langsamer in die gewünschte Richtung, da sie alles neugierig erkunden

4) Welche Aussage zum Treiben von Schweinen ist richtig?
   - **Schweine treibt man tierschutzgerecht am besten in kleinen Gruppen**
   - Ein Schwein lässt sich sehr gut von der Gruppe absondern und treiben
   - Durch den Herdentrieb lassen sich sehr große Schweinegruppen mit mehr als 15 Schweinen am besten treiben

5) Welche Aussage zu Treibhilfen ist richtig?
   - **Die elektrische Treibhilfe sollte als letztes Mittel der Wahl eingesetzt werden und nur unter strengen rechtlichen Voraussetzungen**
   - Spitze Gegenstände wie Forken können auch mit sanftem Druck gut als Treibhilfe eingesetzt werden
   - Peitschen und Stöcke dienen als Verlängerung des Arms und können hilfreiche Treibgegenstände sein

6) Welches sind geeignete und tierschutzgerechte Treibhilfen?
   - **Treibbrett, Treibfahne, Treibpaddel**
   - Treibbrett, Treibfahne, elektrische Treibhilfe
   - Treibpaddel, Treibbrett, Stöcke

Die Elektrobetäubung

1) Welches ist der korrekte Ansatzpunkt für die Elektroden bei der Gehirn- bzw. Kopfdurchströmung?
   - **Beidseits am Ohrgrund**
   - Eine Elektrode auf der Stirn und die zweite im Nacken
   - Beidseits unterhalb der Augen

2) Welche Stromstärke muss bei Schweinen erreicht werden, um eine sichere Elektrobetäubung zu gewährleisten?
   - **1,3A**
   - 1,2A
   - 1,1A

3) Wie viele Sekunden muss die Mindeststromstärke gehalten werden, sodass eine sichere Betäubung beim Mastschwein gewährleistet ist?
   - **4s**
   - 3s
   - 5s

4) Wann darf die elektrische Treibhilfe beim Zutrieb zur Elektrobetäubung eingesetzt werden?
   - **Während der Vereinzelung zur Fixationseinrichtung**
   - Sie ist grundsätzlich verboten
   - Beim Treiben zur Elektrobetäubung darf sie grundsätzlich angewendet werden

5) Welche Aussage zur Elektrobetäubung ist richtig?
   - **Die Elektrobetäubung führt zur Bewusstlosigkeit durch einen epileptischen Anfall im Gehirn**
   - Von den Stromflussparametern kann abgewichen werden, wenn die Schweine sehr ruhig und entspannt sind
   - Die Ansatzpunkte für die Elektroden sind je nach Schweinerasse verschieden

b) **The following are the questions of knowledge for the modules "Handling of pigs" and "Electrical stunning" in Romanian**.

<u>**Manipularea porcilor**</u>

1) Conducerea Porcilor pot avea loc, de asemenea, și fără ajutorul mijloacelor de conducere. Metodele adecvate pentru aceasta sunt
    - **vorbind cu porcii sau bătând din palme**
    - lovirea ușoară a porcilor pe spate cu mâna
    - țipete puternice și mișcări puternice ale mâinilor și picioarelor de către persoana care conduce porcii

2) Ce afirmație despre dispozitivul de propulsie electrică este corectă?
    - **Dispozitivul de propulsie electrică poate fi utilizat o singură dată dacă porcii nu continuă să alerge înainte de a fi separați pentru asomare.**
    - Dispozitivul de propulsie electrică poate fi utilizat în toate zonele fermei (transportor, boxa de deținere, hrănire către CO2 și asomare electrică), dar numai o singură dată pentru fiecare porc.
    - În cazuri excepționale, dispozitivul de propulsie electrică poate fi utilizat de mai multe ori (până la 3 ori) pe porc, dar numai cu puțin timp înainte de asomare

3) Ce afirmație despre porcii care conduc porci este corectă?
    - **conducerea porcilor funcționează cel mai bine într-un mediu calm și lipsit de stres.**
    - Porcii stresați aleargă mai bine în direcția dorită
    - Într-un mediu calm, porcii aleargă mai încet în direcția dorită, deoarece explorează totul cu curiozitate.

4) Ce afirmație despre conducerea porcilor este corectă?
    - **Cel mai bine este ca porcii să fie adunați în grupuri mici, în conformitate cu cerințele privind bunăstarea animalelor.**
    - Este foarte ușor să separi un porc din grup și să-l aduni în turmă.
    - Instinctul de turmă este cel mai bun mod de a aduna grupuri foarte mari de porci, cu mai mult de 15 porci.

5) Ce afirmație despre ajutoarele de deplasare este corectă?
    - **Elicele electrice ar trebui utilizate în ultimă instanță și numai în condiții legale stricte.**
    - Obiectele ascuțite, cum ar fi furculițele, pot fi, de asemenea, folosite ca împunsături cu o presiune ușoară.
    - Biciurile și bastoanele servesc ca o extensie a brațului și pot fi utile pentru a propulsa obiecte.

6) Care sunt ajutoarele de conducere adecvate care sunt potrivite pentru bunăstarea animalelor?
    - **Placă de propulsie, steag de propulsie, vâslă de propulsie**
    - Bord de propulsie, steag de propulsie, ajutor electric de propulsie
    - Vâslă în derivă, placă în derivă, bastoane

Anestezia electrică

1) Care este punctul corect de aplicare a electrozilor în timpul perfuziei creierului sau a capului?
   - **Pe ambele părți, la baza urechii**
   - Un electrod pe frunte și al doilea în gât
   - Pe ambele părți, sub ochi

2) Ce intensitate de curent trebuie să fie atinsă la porci pentru a asigura o asomare electrică sigură?
   - **1,3A**
   - 1,2A
   - 1,1A

3) Câte secunde trebuie menținut curentul minim pentru a asigura asomarea în condiții de siguranță la porcii pentru îngrășare?
   - **4s**
   - 3s
   - 5s

4) Când poate fi utilizat dispozitivul de propulsie electrică pentru asomare electrică?
   - **În timpul separării de dispozitivul de fixare**
   - În general, este interzis
   - Acesta poate fi utilizat atunci când se conduce animalul către dispozitivul de asomare electrică.

5) Ce afirmație despre asomarea electrică este corectă?
   - **Electrocutarea provoacă pierderea cunoștinței din cauza unei crize epileptice la nivelul creierului.**
   - Parametrii fluxului curent pot fi deviați dacă porcii sunt foarte calmi și relaxați.
   - Punctele de aplicare a electrozilor variază în funcție de rasa de porc.

c) **The following are the questions of knowledge for the modules "Handling of pigs" and "Electrical stunning" in English, which have been translated for publication only**.

__Handling of pigs__

1) Moving/driving of pigs can also be done without driving aids. Suitable methods are
    - **talking to the pigs or clapping hands**
    - slightly hitting the pigs with your hand on their backs
    - loud screaming and strong hand and leg movement by the person driving/moving the pigs

2) Which statement about the electric prod is correct?
    - **The electric prod may only be used once if pigs do not continue to walk before being separated in a single file chute**.
    - The electric prod may be used in all areas of the farm (transporter, holding pen, race to CO2 and electric stunning), but only once per pig.
    - The electric prod may be used several times (up to 3 times) on the pig in exceptional cases, but only shortly before stunning.

3) Which statement about moving pigs is correct?
    - **Moving pigs works best in a calm and stress-free environment**.
    - Stressed pigs run better in the desired direction.
    - In a calm environment, pigs run slower in the desired direction because they explore everything curiously.

4) Which statement about driving pigs is correct?
    - **It is best to move pigs in, small, animal welfare friendly groups.**
    - A pig can be very easily separated from the group and then moved.
    - Because of the herd instinct very large groups of pigs with more than 15 pigs can be moved easiest.

5) Which statement about driving aids is correct?
    - **Electric prods should be used as a last resort and only under strict legal conditions.**
    - Pointed objects such as forks can be used with gentle pressure as a driving aid.
    - Whips and sticks serve as an extension of the arm and can be helpful driving aids.

6) Which are suitable and animal welfare friendly driving aids?
    - **Driving board, flag, paddle**
    - Driving board, flag, electric prod
    - Paddle, driving board, sticks

<u>Electrical stunning</u> (translated for publication only)

1) What is the correct attachment point for the electrodes during brain or head-electrical stunning?
    - **On both sides at the base of the ear.**
    - One electrode on the forehead and the second one on the neck.
    - On both sides below the eyes.

2) What current intensity must be reached in pigs to ensure safe electrical stunning?
    - **1.3A**
    - 1.2A
    - 1.1A

3) How many seconds must the minimum current be held so that safe stunning is achieved in fattening pigs?
    - **4s**
    - 3s
    - 5s

4) When may the electric prod be used for driving/moving pigs to the electric stunning device?
    - **During the separation to the fixation device.**
    - It is forbidden in principle.
    - It is allowed when driving the animal to the electric stunning device in general.

5) Which statement about electric stunning is correct?
    - **Electrical stunning causes unconsciousness due to an epileptic seizure in the brain.**
    - The current flow parameters can be deviated from if the pigs are very calm and relaxed.
    - The points of application for the electrodes are different depending on the breed of the pigs.
